# Biosynthesis of Vitamins and Cofactors in Bacterium-Harbouring Trypanosomatids Depends on the Symbiotic Association as Revealed by Genomic Analyses

**DOI:** 10.1371/journal.pone.0079786

**Published:** 2013-11-19

**Authors:** Cecilia C. Klein, João M. P. Alves, Myrna G. Serrano, Gregory A. Buck, Ana Tereza R. Vasconcelos, Marie-France Sagot, Marta M. G. Teixeira, Erney P. Camargo, Maria Cristina M. Motta

**Affiliations:** 1 BAMBOO Team, INRIA Grenoble-Rhône-Alpes, Villeurbanne, France; 2 Laboratoire Biométrie et Biologie Evolutive, Université de Lyon, Université Lyon 1, CNRS, UMR5558, Villeurbanne, France; 3 Laboratório Nacional de Computação Científica, Petrópolis, Rio de Janeiro, Brazil; 4 Department of Parasitology, Institute of Biomedical Sciences, University of São Paulo, São Paulo, Brazil; 5 Virginia Commonwealth University, Richmond, Virginia, United States of America; 6 Instituto de Biofísica Carlos Chagas Filho, Universidade Federal do Rio de Janeiro, Rio de Janeiro, Brazil; Hospital for Sick Children, Canada

## Abstract

Some non-pathogenic trypanosomatids maintain a mutualistic relationship with a betaproteobacterium of the Alcaligenaceae family. Intensive nutritional exchanges have been reported between the two partners, indicating that these protozoa are excellent biological models to study metabolic co-evolution. We previously sequenced and herein investigate the entire genomes of five trypanosomatids which harbor a symbiotic bacterium (**SHTs for Symbiont-Haboring Trypanosomatids**) and the respective bacteria (**TPEs for Trypanosomatid Proteobacterial Endosymbiont**), as well as two trypanosomatids without symbionts (**RTs** **for Regular Trypanosomatids**), for the presence of genes of the classical pathways for vitamin biosynthesis. Our data show that genes for the biosynthetic pathways of thiamine, biotin, and nicotinic acid are absent from all trypanosomatid genomes. This is in agreement with the absolute growth requirement for these vitamins in all protozoa of the family. Also absent from the genomes of RTs are the genes for the synthesis of pantothenic acid, folic acid, riboflavin, and vitamin B_6._ This is also in agreement with the available data showing that RTs are auxotrophic for these essential vitamins. On the other hand, SHTs are autotrophic for such vitamins. Indeed, all the genes of the corresponding biosynthetic pathways were identified, most of them in the symbiont genomes, while a few genes, mostly of eukaryotic origin, were found in the host genomes. The only exceptions to the latter are: the gene coding for the enzyme ketopantoate reductase (EC:1.1.1.169) which is related instead to the Firmicutes bacteria; and two other genes, one involved in the salvage pathway of pantothenic acid and the other in the synthesis of ubiquinone, that are related to Gammaproteobacteria. Their presence in trypanosomatids may result from lateral gene transfer. Taken together, our results reinforce the idea that the low nutritional requirement of SHTs is associated with the presence of the symbiotic bacterium, which contains most genes for vitamin production.

## Introduction

All flagellates of the Trypanosomatidae family (Euglenozoa, Kinetoplastida) are parasites, with hosts among plants, vertebrates (human included) and invertebrates (mainly insects). The non-pathogenic, insect-exclusive parasites contain the largest number of trypanosomatid species, whose most common habitat is the digestive tube of dipterans and hemipterans. Cultures of insect trypanosomatids, also referred to as monoxenics, were first obtained in the 1920s. However, most designated species of these protozoa have not been cultivated and are only known from morphological descriptions recorded in drawings published since the end of the nineteenth century [1].

The modest number of available cultures of insect trypanosomatids is in part due to the difficulties inherent to growing these organisms in artificial media. This is related to the fastidiousness of insect trypanosomatids, which require nutritionally very rich and complex media in order to grow [2]–[4]. The first defined medium for an insect trypanosomatid was published in 1958 [5], as an attempt to cultivate *Crithidia fasciculata*, a species isolated from mosquitoes. The identity of the flagellate, however, cannot be taken at face value because some confusion prevailed at the time (up until today) as concerns the authenticity of the strains and species of insect trypanosomatids.

In most cases, the cultivation of insect trypanosomatids requires all essential amino acids, vitamins of the B-complex, para-aminobenzoate (pABA), inositol, and choline, in addition to purines, glucose, and salts [4]–[5]. Earlier, Newton [6], [7] had described the much simpler nutritional requirements of *Strigomonas oncopelti,* which in addition to the B vitamins needed only methionine, adenine, glucose, and salts for its growth.

Later, it was shown that *S. oncopelti* carries a symbiotic bacterium in its cytoplasm [8], an observation soon extended to some other insect trypanosomatids [9]–[13]. At the current time, six symbiont-bearing trypanosomatids have been identified, which belong to the genera *Angomonas* and *Strigomonas* [13] and harbor a single bacterium per cell [14]. Such symbiotic bacteria are usually referred to as **TPEs** **(Trypanosomatid Proteobacterial Endosymbiont)**, they are vertically transmitted, after a synchronized division with other host cell structures [14]. **Trypanosomatids harboring symbionts** are called **SHTs**, in contrast to **regular trypanosomatids (RTs)** which do not contain symbionts. A considerable amount of information has been gathered about the morphology and cell biology of the host/symbiont association [14]–[17]. From early on, it was suspected that the symbiont was responsible for the enhanced nutritional capabilities of the SHTs, a fact supported by the loss of these capabilities in strains cured of the symbiont (aposymbiotic strains) by chloramphenicol treatment [18]–[20]. Further nutritional studies have shown that, indeed, the requirements of the SHTs are minimal compared to those of RTs [9], [21].

Such nutritional studies suggested that SHTs require neither hemin nor the amino acids that are essential for the growth of RTs. A recent investigation revealed that SHTs have the complete set of genes that code for enzymes of the heme pathway [22]. A similar search on all available metabolic pathways also showed that SHTs have all the gene sequences for the enzymes involved in the synthesis of most essential amino acids [23]. Genomic analyses further revealed that the genes for heme, as well as for the synthesis of essential amino acids, are unequally distributed between the host and the endosymbiont genomes, with most of them being located in the bacterium [22]–[24]. As concerns the need for vitamins by RTs, very little is known mainly because their growth media are very complex, making it difficult to define their specific nutritional requirements. Despite this, various papers addressed indirect aspects of vitamin metabolism [3], [25]–[31]. The development of a defined medium for RTs from insects had initially established that seven vitamins are essential to sustain protozoan growth in culture medium: riboflavin, pantothenic acid, pyridoxamine, folic acid, thiamine, nicotinic acid, and biotin [32]. Studies on the nutritional requirements of insect trypanosomatids did not progress in a satisfactory way, but interestingly demonstrated that SHTs of the genus *Angomonas* are nutritionally much less exigent than RTs [9]. Thus, while the autotrophy of SHTs for most of the B vitamins was evidenced, nothing was known about pathways for the synthesis of other vitamins. Furthermore, any direct evidence of the symbiont contribution to the vitamin synthetic capabilities of the host trypanosomatid was missing.

The acquisition of metabolic capabilities through a mutualistic symbiosis with bacteria is widespread among eukaryotes. The sap-feeding insects are well studied examples of this [33]–[35]. The great majority of these associations enables the synthesis of the essential amino acids not available in the poor diet of the insect hosts. In some cases, the bacterial symbionts are able to produce vitamins of the B complex and cofactors. Such is the case of the endosymbiont, *Wigglesworthia glossinidia*, of the tsetse fly and also *Candidatus* Baumannia cicadellinicola, an endosymbiont of the sharpshooter [36], [37]. The latter is in a dual bacterial symbiosis, where one partner (*Ca.* Sulcia muelleri) supplies amino acids to the host whereas the other (*Ca.* B. cicadellinicola) provides vitamins and cofactors. This makes the sharpshooter less nutritionally exigent [37].

In recent studies, we reported on the sequencing of the entire genomes of five species of TPEs [24] and we also annotated the proteins of two SHT species and their respective symbionts [38]. Moreover, we sequenced to a draft-level the genomes of the five host species as well as of two RTs [23]. In this paper, we analyze these genomes for the presence of genes involved in the synthesis of vitamins. The participation of both host and symbiont in the production of vitamins is presented and discussed in association with previous data on the nutritional requirements of RTs and SHTs. In order to get a broader view, we compared our findings with other trypanosomatids and bacteria from the Alcaligenaceae family based on KEGG [39].

## Materials and Methods

### 2.1. Analyzed organisms and their genome sequences

The genomes of the following SHTs and of the respective symbionts were examined: *Strigomonas oncopelti* TCC290E (accession number AUXK00000000), *S. culicis* TCC012E (AUXH00000000), *S. galati* TCC219 (AUXN00000000), *Angomonas deanei* TCC036E (AUXM00000000), and *A. desouzai* TCC079E (AUXL00000000) [23]. Their corresponding symbionts are referred to as: “*Candidatus* Kinetoplastibacterium oncopeltii”, “*Ca*. K. blastocrithidii”, “*Ca*. K. galatii”, “*Ca*. K. crithidii”, and “*Ca*. K. desouzaii” [13]. The endosymbiont genomes were finished to a closed circle as previously described [24].

The genomes of two RTs were also analyzed: *Herpetomonas muscarum* TCC001E (AUXJ00000000) and *Crithidia acanthocephali* TCC037E (AUXI00000000) [23].

### 2.2. Gene discovery and annotation

Initially, the trypanosomatid genes were discovered and mapped to metabolic pathways using ASGARD [40], using as reference the UniRef100 [41] and KEGG [39] databases. The identified segments of DNA were then extracted from the genomes and manually curated for completion and proper location of start and stop codons by using the GBrowse genome browser [42]. Putative sequence functions were confirmed by domain searches against the NCBI's CDD (conserved domain database) [43]. For each enzyme characterized in this work, corresponding putative orthologous genes from all domains of life were collected from the public databases by BLAST search (E-value cutoff of 1e-10, maximum of 10,000 matches accepted) against the full NCBI NR protein database, collecting sequences from taxonomic groups as widespread as possible and keeping one from each species (or genus, if the tree was too large) for subsequent phylogenetic analysis. Only sequences that were complete and aligned along at least 75% of the length of the query were selected.

All trypanosomatid genes characterized in this study have been submitted to NCBI's GenBank; accession numbers are available in Table S2. All endosymbiont genes analyzed here have been previously sequenced [24]; gene identifiers are available in Table S3.

For comparison, we used in our analyses the genome annotations of trypanosomatids (*Trypanosoma brucei, T. cruzi, Leishmania major, L. infantum, L. donovani, L. mexicana, L. braziliensis*) and bacteria from the Alcaligenaceae family (*Bordetella pertussis* Tohama II, *B. pertussis* CS, *B. pertussis* 18323, *B. parapertussis* 12822, *B. parapertussis* Bpp5, *B. bronchiseptica* RB50, *B. bronchiseptica* MO149, *B. bronchiseptica* 253, *B. petrii*, *B. avium*, *Achromobacter xylosoxidans*, *Taylorella equigenitalis* MCE9, *T. equigenitalis* ATCC 35865, *T. asinigenitalis*, *Pusillimonas* sp. T7-7, *Advenella kashmirensis*) available in KEGG [39]. However, care should be taken since these data may lack manual curation. As concerns information on metabolic pathways, we used KEGG [39] and MetaCyc [44].

### 2.3. Phylogenetic analyses

All analyses were performed at the protein sequence level. Sequences were aligned by using MUSCLE [45] and phylogenetic inferences were performed by the maximum likelihood (ML) method using RAxML v. 7.2.8 [46] and the WAG amino acid substitution model [47], with four gamma-distributed substitution rate heterogeneity categories and empirically determined residue frequencies (model PROTGAMMAWAGF). Each alignment was submitted to bootstrap analysis with 100 pseudo-replicates. We also performed phylogenetic inferences by the neighbor joining (NJ) method using the seqboot and neighbor programs from PHYLIP v. 3.69 [48] and RAxML v. 7.2.8 for the distance matrix calculation (in order to use the same amino acid substitution model) and for drawing the bootstrap support values (100 replicates) in the NJ tree. Trees were initially drawn and formatted using TreeGraph2 [49] and Dendroscope [50], with subsequent cosmetic adjustments performed with the Inkscape vector image editor (http://inkscape.org). CodonW [51] was used to perform correspondence analyses of codon usage and to calculate codon adaptation index scores for the candidate HGT genes using an endosymbiont gene as a negative control.

## Results and Discussion

We analyzed the genomes of five species of SHTs and of their TPEs for the presence/absence of genes from the metabolic pathways for essential vitamin synthesis. The genomes of two RTs, *C. acanthocephali* and *H. muscarum*, were examined in detail, however these data do not fully represent the genomic diversity of insect trypanosomatids in general. Indeed, the enormous diversity present in the Trypanosomatidae family is sometimes not fully appreciated, leading to apparent conflicts in the interpretation of metabolic data, as happened with the early studies on the nutrition of *Crithidia* species. Data on the nutritional requirements of *C. fasciculata* strongly disagreed with those obtained for *C. oncopelti* (recently renamed as *Strigomonas oncopelti*) as concerns the necessity for amino acids and vitamins which is quite different for both organisms [5], [6]. Many years elapsed until it was realized that these organisms were quite distinct phylogenetically, and in fact belonged to different genera [13]. It became clear that *S. oncopelti*, as well as other trypanosomatids which were later isolated, carried a bacterial symbiont that probably endowed the host with enhanced biosynthetic capabilities. According to our present data, these extra nutritional capabilities largely result from the contribution of the endosymbiont to the metabolism of their trypanosomatid hosts as will be discussed here when analyzing vitamin biosynthesis in SHTs.

### 3.1. Autotrophy of SHTs for the synthesis of riboflavin, pantothenic acid, vitamin B_6_ and folic acid

**Figure 1:**
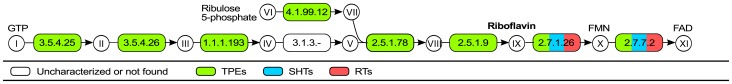
Biosynthesis of riboflavin and FAD. **Metabolites - I**: Guanosine 5'-triphosphate; **II**: 2,5-Diamino-6-(5-phospho-D-ribosylamino)pyrimidin-4(3H)-one; **III**: 5-Amino-6-(5'-phosphoribosylamino)uracil; **IV**: 5-Amino-6-(5'-phospho-D-ribitylamino)uracil; **V**: 5-Amino-6-(1-D-ribitylamino)uracil; **VI**: D-Ribulose 5-phosphate; **VII**: 2-Hydroxy-3-oxobutyl phosphate; **VIII**: 6,7-Dimethyl-8-(D-ribityl)lumazine; **IX**: Riboflavin; **X**: Flavin mononucleotide; **XI**: Flavin adenine dinucleotide **Enzymes - 3.5.4.25**: GTP cyclohydrolase II; **3.5.4.26**: diaminohydroxyphosphoribosylaminopyrimidine deaminase; **1.1.1.193**: 5-amino-6-(5-phosphoribosylamino)uracil reductase; **3.1.3.-**: Phosphoric monoester hydrolases; **4.1.99.12**: 3,4-dihydroxy 2-butanone 4-phosphate synthase; **2.5.1.78**: 6,7-dimethyl-8-ribityllumazine synthase; **2.5.1.9**: riboflavin synthase; **2.7.1.26**: riboflavin kinase; **2.7.7.2**: FAD synthetase.

**3.1.1. Riboflavin (Vitamin B_2_).** Riboflavin is essential for the growth of RTs, as well as for the aposymbiotic strains of SHTs [3], [5], [9], but not for the symbiont-carrying strains of SHTs, which are autotrophic for this vitamin [6], [9], [32]. Riboflavin is synthesized from guanosine 5'-triphosphate (GTP) and ribulose 5'-phosphate (Figure 1), and is the precursor for the essential flavin cofactors of redox reactions: FMN (flavin mononucleotide) and FAD (flavin adenine dinucleotide) [52]. The genomes of SHTs and RTs have none of the genes for the enzymes involved in riboflavin synthesis. On the other hand, TPEs have all the genes responsible for such synthesis, except for a poorly characterized step in the pathway, probably involving a phosphoric monoester hydrolase (Figure 1, IV-V). However, it is uncertain which enzyme is responsible for this dephosphorylation process although it was suggested that a phosphatase of low substrate specificity might be involved [52], [37]. Bacteria from the Alcaligenaceae family have all the enzymes for the synthesis of riboflavin as is the case for TPEs, missing only the uncharacterized one (Figure S1). Since SHTs do not require riboflavin, it can be assumed that the dephosphorylation reaction is catalyzed by any of a cohort of phosphatases of broad substrate range.

Further along the riboflavin biosynthetic pathway, it can be seen that all trypanosomatids, with or without symbionts, have the genes for the conversion of riboflavin into FMN and of the latter into FAD. Those genes are also present in other trypanosomatids (Figure S1). It is worth considering that in SHTs the presence of such genes in the trypanosomatid host may be related to the control of the production of FMN and FAD. FMN acts as a coenzyme in oxidative enzymes, including NADH dehydrogenase while FAD forms the prosthetic group of certain oxidases, both serving as electron carriers. Recently, we proposed that the presence of the symbiont influences the energetic metabolism of *A. deanei* (unpublished data). This analysis reinforces this idea and reveals that, thanks to the genes of the symbiont, SHTs are fully capable of riboflavin synthesis, corroborating the nutritional data that point to this vitamin as unnecessary for the growth of SHTs, although indispensable for the growth of RTs [5], [9], [32].

**Figure 2:**
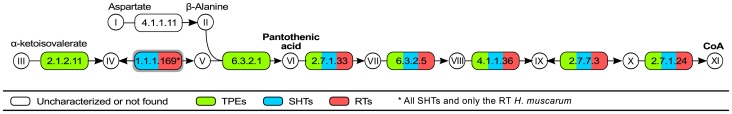
Biosynthesis of pantothenic acid and coenzyme A. Enzymes surrounded by a gray box were possibly acquired through horizontal transfer from Bacteria to trypanosomatids (see main text). **Metabolites - I**: Aspartate; **II**: β-Alanine; **III**: α-ketoisovalerate; **IV**: 2-Dehydropantoate; **V**: Pantoate; **VI**: Pantothenic acid; **VII**: D-4'-Phosphopantothenate; **VIII**: (R)-4'-Phosphopantothenoyl-L-cysteine; **IX**: Pantetheine 4'-phosphate; **X**: Dephosphocoenzyme A; **XI**: Coenzyme A. **Enzymes -** **4.1.1.11**: aspartate 1-decarboxylase; **2.1.2.11**: 3-methyl-2-oxobutanoate hydroxymethyltransferase; **1.1.1.169**: ketopantoate reductase; **6.3.2.1**: pantoate--beta-alanine ligase; **2.7.1.33**: pantothenate kinase; **6.3.2.5**: phosphopantothenate-cysteine ligase; **4.1.1.36**: phosphopantothenoylcysteine decarboxylase; **2.7.7.3**: pantetheine-phosphate adenylyltransferase; **2.7.1.24**: dephospho-CoA kinase.

**3.1.2. Pantothenic acid (Vitamin B_5_).** Early nutritional studies considered pantothenic acid as an absolute requirement for the growth of trypanosomatids [3], [5]. Later reports confirmed these observations, but showed also that pantothenate is not at all necessary for the cultivation of SHTs such as *S. oncopelti* and *A. deanei* [6], [9], [32]. Bacteria synthesize coenzyme A (CoA) via pantothenic acid from aspartate and α-ketoisovalerate (Figure 2), while CoA is an acyl carrier required for a multitude of reactions for both biosynthetic and degradation pathways [53]. The CoA biosynthetic route requires nine enzymes: four to synthesize pantothenic acid (Figure 2, I-VI) and five to produce CoA (Figure 2, VI-XI).

As concerns the first half, the enzyme aspartate 1-decarboxylase (EC:4.1.1.11), required for the conversion of aspartate into β-alanine (Figure 2, I-II), was not identified in TPEs, SHTs nor RTs. Moreover, the two latter groups possess the enzymes to catalyze the synthesis of β-alanine from malonyl-CoA. TPEs have two enzymes responsible for the synthesis of pantothenic acid (3-methyl-2-oxobutanoate hydroxymethyltransferase EC:2.1.2.11; and pantoate--beta-alanine ligase EC:6.3.2.1; Figure 2, III-IV and V-VI) which were not found in SHTs nor in RTs. Moreover, the genes necessary to convert pyruvate into α-ketoisovalerate (one precursor of this biosynthetic pathway) were only identified in TPEs, but neither in SHTs nor in RTs [23]. These steps take part in the biosynthetic route of valine. The remaining step is the production of pantoate mediated by ketopantoate reductase (EC:1.1.1.169, Figure 2, VI-V), that participates exclusively in this pathway and was identified in all the SHTs analyzed and also in *H. muscarum*. In SHTs, its presence would be meaningful for the synthesis of pantothenic acid complemented by the TPEs, however the presence of this gene in *H. muscarum* is puzzling since RTs lack the remaining genes of the pantothenic acid biosynthetic pathway. As discussed in detail below in Section 3.4 (Phylogenetic Analyses), this gene is likely a relic from a past lateral gene transfer event from a bacterium to a common ancestor of the Trypanosomatidae family.

Other trypanosomatids available in KEGG lack all the enzymes for the production of pantothenic acid. Most bacteria from the Alcaligenaceae family have all the machinery for this biosynthesis (*Bordetella* spp. and *A. xylosoxidans*) while *Pusillimonas* sp. and *A. kashmirensis* lack only enzyme EC:4.1.1.11. On the other hand, *Taylorella* spp, which have the most reduced genomes, lack the first 3 steps (Figure S1).

The second half of the pathway, which is the production of CoA from pantothenic acid, is accomplished by TPEs, SHTs, and RTs. In SHTs, we can assume that CoA formation is optimized by TPEs. This coenzyme is related to the synthesis and oxidation of fatty acids and oxidation of pyruvate in the Krebs cycle, which is directly associated with amino acid production. Recently, our genomic searches showed that the symbionts of the trypanosomatids contain the genes coding for enzymes that complete the host pathways for the synthesis of essential amino acids [23].

Based on those findings, the association of host/symbiont makes SHTs autotrophic for pantothenic acid production [6], [9], [32], whereas RTs depend on an exogenous source of this vitamin since they lack some genes for the synthesis of pantothenic acid [3], [5].

**3.1.3. Pyridoxal, pyridoxine and pyridoxamine (Vitamin B_6_).** Vitamin B_6_ refers collectively to pyridoxal, pyridoxine, pyridoxamine and their corresponding phosphate esters. Its catalytically active forms are pyridoxal 5’-phosphate (PLP) and pyridoxamine 5′-phosphate (PMP) [54]. This vitamin is essential for all organisms while PLP is an extremely versatile coenzyme necessary for over 100 enzymatic reactions, predominantly in the metabolism of amino acids [54], [55]. Pyridoxal or pyridoxamine was described as an essential growth factor for RTs, as well as for the aposymbiotic strain of *A. deanei* [5], [19]. On the other hand, it was identified as not required by SHTs despite the fact that its presence doubled the growth rate of *S. oncopelti* [6], [9].

**Figure 3:**
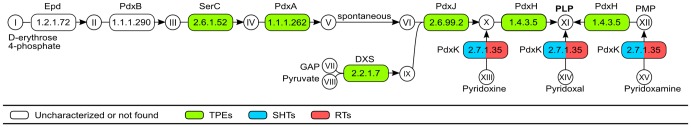
Biosynthesis of vitamin B6. **Metabolites - I**: D-Erythrose 4-phosphate; **II**: 4-Phospho-D-erythronate; **III**: 2-Oxo-3-hydroxy-4-phosphobutanoate; **IV**: 4-phospho-hydroxy-L-threonine; **V**: 2-amino-3-oxo-4-phosphonooxybutyrate; **VI**: 3-Amino-2-oxopropyl phosphate; **VII**: D-glyceraldehyde 3-phosphate; **VIII**: pyruvate; **IX**: 1-deoxy-D-xylulose 5-phosphate; **X**: Pyridoxine phosphate; **XI**: Pyridoxal 5’-phosphate (PLP); **XII**: Pyridoxamine phosphate; **XIII**: Pyridoxine; **XIV**: Pyridoxal; **XV**: Pyridoxamine. **Enzymes -** **1.2.1.72**: D-erythrose 4-phosphate dehydrogenase; **1.1.1.290**: erythronate-4-phosphate dehydrogenase; **2.6.1.52**: phosphoserine aminotransferase; **1.1.1.262**: 4-hydroxythreonine-4-phosphate dehydrogenase; **2.2.1.7**: 1-deoxyxylulose-5-phosphate synthase; **2.6.99.2**: pyridoxine 5-phosphate synthase; **1.4.3.5**: pyridoxamine 5'-phosphate oxidase; **2.7.1.35**: pyridoxal kinase.

As shown in Figure 3, the precursors for the *de novo* biosynthesis of PLP are D-erythrose-4-phosphate, glyceraldehyde-3-phosphate (GAP), and pyruvate [54]. The genomes of RTs and SHTs have none of the enzymes for the synthesis of PLP, whereas TPEs have most of them, except for the first two steps mediated by the enzymes D-erythrose 4-phosphate dehydrogenase and erythronate-4-phosphate dehydrogenase (Epd EC:1.2.1.72 and PdxB EC:1.1.1.290, respectively), which convert D-erythrose-4-phosphate into 2-Oxo-3-hydroxy-4-phosphobutanoate (Figure 3, I-III). Since SHTs are autotrophic for PLP, these steps might be mediated by other distinct and unknown enzymes, or TPEs might use a precursor different from D-erythrose-4-phosphate. These same two steps are also missing in bacteria from the Alcaligenaceae family (Figure S1). The gene coding for Epd shares a high sequence similarity with *gap*A (gene coding for glyceraldehyde 3'-phosphate dehydrogenase, involved in glycolysis). Based on mutant essays, GapA was shown to be able to replace the Epd activity under certain conditions [56]. Since Epd was the only enzyme not identified in this pathway in *Ca.* B. cicadellinicola (endosymbiont of the sharpshooter), GapA was suggested as a candidate [37]. GapA is present in TPEs and also in all bacteria from the Alcaligenaceae family.

On the other hand, the RT and SHT genomes have the gene for pyridoxal kinase (EC:2.7.1.35), which converts pyridoxal, pyridoxine, and pyridoxamine into their respective phosphate esters (salvage pathway), including PLP, the active principle of the B_6_ complex. However, they lack the oxidase responsible for the interconversion of the different forms of vitamin B_6_ (see PdxH in Figure 3). Considering other trypanosomatids, there is no enzyme involved in this biosynthetic pathway, only the kinase above mentioned which is involved in the salvage pathway of vitamin B_6_ (Figure S1).

Together, these findings underline the auxotrophy of RTs and the autotrophy of SHTs for the B_6_ complex [5], [6], [9], [21], [32]. Furthermore, PLP is an active coenzyme that acts especially on the metabolism of amino acids. This can be directly related to the low nutritional requirement of SHTs since essential amino acids are synthesized by the symbiotic bacterium [23].

**Figure 4:**
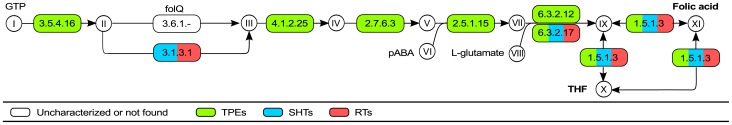
Biosynthesis of folic acid. **Metabolites - I**: Guanosine 5'-triphosphate; **II**: 7,8-Dihydroneopterin 3'-triphosphate; **III**: Dihydroneopterin; **IV**: 2-Amino-4-hydroxy-6-hydroxymethyl-7,8-dihydropteridine; **V**: 2-Amino-7,8-dihydro-4-hydroxy-6-(diphosphooxymethyl)pteridine; **VI**: para-aminobenzoate; **VII**: Dihydropteroate; **VIII:** L-glutamate; **IX**: Dihydrofolate; **X**: Tetrahydrofolate; **XI**: Folic acid. **Enzymes -** **3.5.4.16**: GTP cyclohydrolase I; **3.1.3.1**: alkaline phosphatase; **3.6.1.-**: Hydrolase acting on acid anhydrides in phosphorus-containing anhydrides; **4.2.1.25**: dihydroneopterin aldolase; **2.7.6.3**: 2-amino-4-hydroxy-6-hydroxymethyldihydropteridine diphosphokinase; **2.5.1.15**: dihydropteroate synthase; **6.3.2.12**: dihydrofolate synthase; **6.3.2.17**: folylpolyglutamate synthase; **1.5.1.3**: dihydrofolate reductase.

**3.1.4. Folic acid (Vitamin B_9_).** Folic acid is considered an essential growth factor for RTs [3], [5] despite the prevailing difficulties in defining essential requirements in complex culture media. However, after using a defined growth medium, it was confirmed that folic acid is indeed an absolute requirement for the growth of regular trypanosomatids [32]. Conversely, it was shown that in SHTs such as *S. oncopelti* [6] and *A. desouzai* [31], growth occurs in total absence of folic acid. The standard pathway for the synthesis of folic acid is shown in Figure 4. Folates are composed of pterin, para-aminobenzoate (pABA), and L-glutamate moieties. Pterin is synthesized from GTP (guanosine 5'-triphosphate), whereas pABA is obtained from chorismate [57].

The genomes of all the TPEs examined carry the genes for the conversion of GTP, pABA, and L-glutamate into folate and tetrahydrofolate (THF), except for the step that removes the triphosphate motif of 7,8-dihydroneopterin triphosphate to produce dihydroneopterin (Figure 4, II-III). This step was for long unknown. It was recently shown in bacteria and in plants that this reaction is performed by an enzyme from the Nudix family called FolQ (or NudB, dATP pyrophosphohydrolase EC:3.6.1.-) [58], [59]. The corresponding gene is not assigned in any bacteria of the Alcaligenaceae family, and it is not possible, based only on a sequence similarity search, to find a candidate for the step. *Ca.* B. cicadellinicola, an endosymbiont of the sharpshooter, lacks only this gene for folate synthesis [37]. On the other hand, Nudix proteins are found in TPEs as well as in the members of the Alcaligenaceae family. Alcaligenaceae bacteria have the other genes for the conversion of GTP, pABA, and L-glutamate into folate and tetrahydrofolate, but most *Bordetella* spp. lack the first step of this pathway (GTP cyclohydrolase EC:3.5.4.16, Figure S1). In KEGG, we also found an alkaline phosphatase (EC:3.1.3.1) of broad spectrum as an option for the missing step (Figure 4, II-III), which is present in SHTs and RTs but not in TPEs.

Further down in the THF biosynthetic pathway, the genes coding for the last two enzymes of the folic acid and THF synthesis, folylpolyglutamate synthase (EC:6.3.1.17) and dihydrofolate reductase (EC:1.5.1.3), are present in the TPE, SHT, and RT genomes. They are also present in *Trypanosoma* and *Leishmania* spp. (Figure S1), and the latter genus is able to salvage folate and unconjugated pteridines from their hosts [60].

As mentioned above, pABA has been described as a nutritional requirement for *S. oncopelti* [6], [7]. Conversely, this metabolite is absent in the minimal medium for *C. fasciculata* [5], however it is interesting to observe that folate is required in this case. Since pABA is an intermediate for folic acid biosynthesis, it makes perfect sense that the uptake of folate from the diet dispenses the need for pABA. Its synthesis from chorismate requires pabAB (aminodeoxychorismate synthase EC:2.6.1.85) and pabC (aminodeoxychorismate lyase EC:4.1.3.38) [57]. These enzymes were not identified in TPEs, SHTs or RTs. Those steps are found in the Alcaligenaceae bacteria except for *Taylorella* spp., and they are absent in *Leishmania* and *Trypanosoma* spp. The inability of SHTs and TPEs to produce pABA agrees with the described need for this metabolite in the minimal medium of *S. oncopelti*; in other words, TPEs would be able to synthesize folate provided pABA is available. This corroborates the fact that folic acid was considered a nutritional requirement for *A. deanei* when pABA was not supplied [9].

As a result, TPEs potentially have the enzymatic machinery for folate synthesis but probably require an exogenous source of pABA, corroborating the fact that SHTs are autotrophic for folic acid [6], [7], [21], [31].

### 3.2. Auxotrophy of trypanosomatids for thiamine, nicotinic acid and biotin

**3.2.1. Thiamine (Vitamin B_1_).** Thiamine is an essential growth factor for RTs, as well as for SHTs [5], [6], [9]. Studies on the SHT requirement for thiamine were first performed with *S. oncopelti* [6] and only later were extended to *Angomonas* spp. [9]. In both cases, thiamine was found to be an essential growth factor. This indicated that all or some of the genes for the biosynthesis of thiamine were missing from the genomes of both hosts and symbionts, which is in agreement with the genomic analysis performed in this work. Thiamine is particularly important for carbohydrate metabolism and its pathway involves the separate synthesis of thiazole and pyrimidine which are then coupled to form thiamine diphosphate (thiamine-PPi), which is the biologically active form of vitamin B_1_ [57].

**Figure 5:**
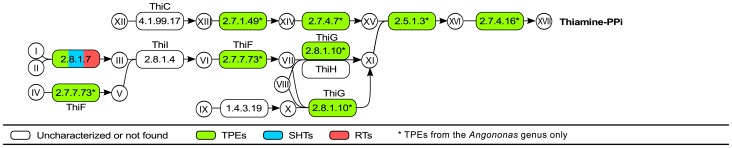
Biosynthesis of thiamine. **Metabolites - I**: L-Cysteine; **II**: a [ThiI sulfur-carrier protein]-L-cysteine; **III**: a [ThiI sulfur-carrier protein]-S-sulfanylcysteine; **IV**: a ThiS sulfur carrier protein; **V**: a carboxy-adenylated-[ThiS sulfur-carrier protein]; **VI**: Thiamine biosynthesis intermediate 5; **VII**: a thiocarboxy-adenylated-[ThiS-protein]; **VIII**: L-Tyrosine; **IX**: Glycine; **X**: Iminoglycine; **XI**: 4-Methyl-5-(2-phosphoethyl)-thiazole; **XII**: 5'-Phosphoribosyl-5-aminoimidazole; **XIII**: 4-Amino-5-hydroxymethyl-2-methylpyrimidine; **XIV**: 4-Amino-2-methyl-5-phosphomethylpyrimidine; **XV**: 2-Methyl-4-amino-5-hydroxymethylpyrimidine diphosphate; **XVI**: Thiamine monophosphate; **XVII**: Thiamine diphosphate. **Enzymes - 2.8.1.7**: cysteine desulfurase; **2.7.7.73**: sulfur carrier protein ThiS adenylyltransferase; **2.8.1.4**: thiamine biosynthesis protein ThiI; **1.4.3.19**: glycine oxidase; **2.8.1.10**: thiamine biosynthesis ThiG; **4.1.99.19**: thiamine biosynthesis ThiH; **4.1.99.17**: thiamine biosynthesis protein ThiC; **2.7.1.49**: hydroxymethylpyrimidine kinase; **2.7.4.7**: phosphomethylpyrimidine kinase; **2.5.1.3**: thiamine-phosphate pyrophosphorylase; **2.7.4.16**: thiamine-monophosphate kinase.

Most genes related to the biosynthesis of thiamine are present only in the TPEs of *Angomonas* and totally absent from the genomes of RTs, SHTs, as well as from the TPEs from *Strigomonas* (Figure 5). However, even the symbionts of *Angomonas* lack the genes for key enzymes such as cysteine desulfurase (EC 4.1.99.17), thiamine biosynthesis protein ThiI (EC:2.8.1.4), and glycine oxidase (EC:1.4.3.19) that mediate the initial steps of any of the pathways leading to the synthesis of thiamine. Only the gene for cysteine desulfurase (EC:2.8.1.7) was found in all genomes including those of SHTs and RTs, but its presence may be related to its participation in the sulfur relay system. A few steps of the thiamine biosynthesis are missing in bacteria from the Alcaligenaceae family while the pathway is totally absent in *Leishmania* and *Trypanosoma* spp. (Figure S1). The genomic profile of RTs, SHTs, and TPEs is thus in perfect agreement with the absolute need of thiamine for the growth of trypanosomatids in general.

**Figure 6:**
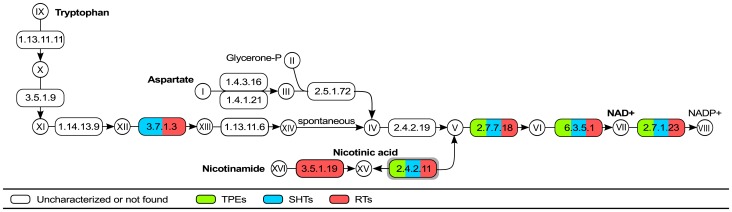
Biosynthesis of nicotinic acid and NAD. Enzymes surrounded by a gray box were possibly acquired through horizontal transfer from Bacteria to trypanosomatids (see main text). **Metabolites - I**: Aspartate**; II:** Glycerone-phosphate**; III:** Iminoaspartate**; IV:** Quinolinate**; V:** Nicotinate D-ribonucleotide**; VI:** Deamino-NAD+**; VII:** Nicotinamide adenine dinucleotide**; VIII:** Nicotinamide adenine dinucleotide phosphate**; IX:** Tryptophan; **X**: L-Formylkynurenine**; XI:** L-Kynurenine**; XII:** 3-Hydroxy-L-kynurenine**; XIII:** 3-Hydroxyanthranilate**; XIV:** 2-Amino-3-carboxymuconate semialdehyde**; XV:** Nicotinic acid; **XVI:** Nicotinamide. **Enzymes -** **1.4.3.16:** L-aspartate oxidase**; 1.4.1.21:** aspartate dehydrogenase**; 2.5.1.72:** quinolinate synthase**; 2.4.2.19:** nicotinate-nucleotide diphosphorylase**; 2.7.7.18:; 6.3.5.1:** NAD+ synthase**; 2.7.1.23:** NAD+ kinase**; 1.13.11.11:** tryptophan 2,3-dioxygenase**; 3.5.1.9:** arylformamidase**; 1.14.13.9:** kynurenine 3-monooxygenase**; 3.7.1.3** kynureninase**; 1.13.11.6:** 3-hydroxyanthranilate 3,4-dioxygenase; **2.4.2.11**: nicotinate phosphoribosyltransferase (recently transferred to EC6.3.4.21); **3.5.1.19**: nicotinamidase.

**3.2.2. Nicotinic acid (Vitamin B_3_).** Nicotinic acid is also essential for the growth of any kind of trypanosomatid, with or without endosymbionts [3], [5], [6], [9], [19], [21], [32]. The precursors for the *de novo* biosynthesis of nicotinamide adenine dinucleotide (NAD) are aspartate in prokaryotes and tryptophan in prokaryotes and eukaryotes [61], [62]. However, TPEs, SHTs, and RTs do not possess the enzymatic machinery for any of these processes. On the other hand, the genes responsible for the conversion of nicotinic acid into NAD+ and NADP+ are present in the genomes of all the TPEs, SHTs, and RTs examined (Figure 6). Interestingly, nicotinamidase (EC:3.5.1.19, Figure 6, XVI-XV), a key enzyme of this salvage pathway that catalyzes the conversion of nicotinamide to nicotinic acid, has been recently biochemically and functionally characterized in *L. infantum* [63]. Based on this sequence, we were able to identify candidates for this gene in the two RTs analyzed in the present study and in *Trypanosoma* and *Leishmania* spp., however not in SHTs or TPEs (Figure S1). This is in agreement with the fact that nicotinamide is frequently described in the minimal media of RTs [5], since it can be converted into nicotinic acid. There is also agreement that, once nicotinic acid is provided, all trypanosomatids are able to synthesize NAD, the essential coenzyme for the redox reactions of any living cell. As concerns the RT species, since they have the gene coding for nicotinamidase, they are also able to grow in culture medium containing only nicotinamide.

**3.2.3. Biotin (Vitamin B_7_).** The need for biotin was demonstrated for RTs as well as for *A. deanei* [3], [5]. In the case of *S. oncopelti*, it was described as a non-essential vitamin, although its growth rate doubled with the addition of biotin to the media [6].

**Figure 7:**
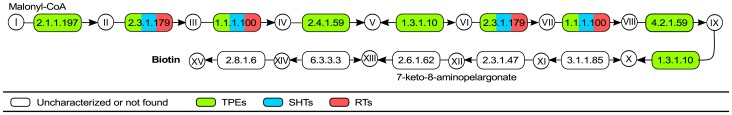
Biosynthesis of biotin. **Metabolites - I**: malonyl-CoA; **II**: malonyl-CoA methyl ester; **III**: a 3-oxo-glutaryl-[acp] methyl ester; **IV**: a 3-hydroxyglutaryl-[acp] methyl ester; **V**: an enoylglutaryl-[acp] methyl ester; **VI**: a glutaryl-[acp] methyl ester; **VII**: a 3-oxo-pimelyl-[acp] methyl ester; **VIII**: a 3-hydroxypimelyl-[acp] methyl ester; **IX**: an enoylpimelyl-[acp] methyl ester; **X**: a pimelyl-[acp] methyl ester; **XI**: a pimelyl-[acp]; **XII**: 7-keto-8-aminopelargonate; **XIII**: 7,8-diaminopelargonate; **XIV**: dethiobiotin; **XV**: biotin. **Enzymes -** **2.1.1.197**: malonyl-CoA methyltransferase; **2.3.1.180**: β-ketoacyl-acyl carrier protein synthase III; **1.1.1.100**: 3-oxo-acyl-[acyl-carrier-protein] reductase; **2.4.1.59**: 3-hydroxy-acyl-[acyl-carrier-protein] dehydratase; **1.3.1.10**: enoyl-[acyl-carrier-protein] reductase; **2.3.1.41**: β-ketoacyl-ACP synthase I; **3.1.1.85**: pimeloyl-[acp] methyl ester esterase; **2.3.1.47**: 8-amino-7-oxononanoate synthase; **2.6.1.62**: 7,8-diaminopelargonic acid synthase; **6.3.3.3**: dethiobiotin synthetase; **2.8.1.6**: biotin synthase.

Malonyl-CoA has been recently described as the precursor of the pimeloyl moiety of biotin in *Escherichia coli* by a modified fatty acid synthetic pathway [64]. The late steps of the biotin biosynthetic pathway (Figure 7, XI-XV) are responsible for forming the two rings in the structure of this coenzyme. The trypanosomatid genomes have a few genes of the upper part of the pathway, also identified in *Trypanosoma* and *Leishmania* spp. (Figure 7, Figure S1). On the other hand, the symbionts possess the genes for the first nine steps of the pathway starting from malonyl-CoA, but lack the remaining ones (Figure 7). Bacteria from the Alcaligenaceae family have most of the genes for the entire pathway (Figure S1).

This indicates that neither RTs nor SHTs are capable of biotin synthesis. The growth of *S. oncopelti* in the absence of exogenous biotin is thus puzzling, unless this protozoan synthesizes biotin via a distinct, unusual route. This would be the most probable alternative if the nutritional autotrophy of *S. oncopelti* is confirmed, which has not been the case so far.

### 3.3. Other cofactors

Cofactors such as lipoic acid are produced by SHTs and RTs but not by TPEs. Conversely, the cobalamin (vitamin B_12_) and menaquinone synthetic pathways are absent in all trypanosomatids and symbionts. Interestingly, the ubiquinone biosynthetic route is present in all RTs and SHTs as well as in the TPEs from the *Strigomonas* genus but absent in the TPEs from the *Angomonas* genus.

Ubiquinone functions as an electron carrier in membranes and is composed of a benzoquinone ring and an isoprene side chain which varies in the number of subunits in different organisms [65]. In *L. major*, the ubiquinone ring synthesis has been described as having either acetate (via chorismate as in prokaryotes) or aromatic amino acids (as in mammalian cells) as precursor [65].

**Figure 8:**
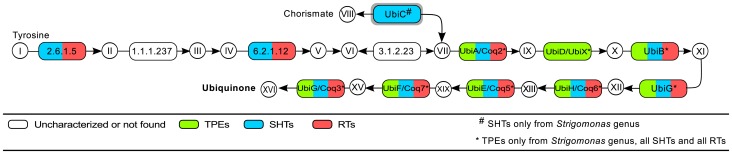
Ubiquinone biosynthesis. Enzymes surrounded by a gray box were possibly acquired through horizontal transfer from Bacteria to trypanosomatids (see main text). **Metabolites - I**: L-Tyrosine; **II**: 4-Hydroxyphenylpyruvate; **III**: 4-Hydroxyphenyllactate; **IV**: 4-Coumarate; **V**: 4-Coumaroyl-CoA; **VI**: 4-Hydroxybenzoyl-CoA; **VII**: 4-Hydroxybenzoate; **VIII**: Chorismate; **IX**: 4-Hydroxy-3-polyprenylbenzoate; **X**: 2-Polyprenylphenol; **XI**: 2-Polyprenyl-6-hydroxyphenol; **XII**: 2-Polyprenyl-6-methoxyphenol; **XIII**: 2-Polyprenyl-6-methoxy-1,4-benzoquinone; **XIV**: 2-Polyprenyl-3-methyl-6-methoxy-1,4-benzoquinone; **XV**: 2-Polyprenyl-3-methyl-5-hydroxy-6-methoxy-1,4-benzoquinone; **XVI**: Ubiquinone. **Enzymes -** **2.6.1.5**: tyrosine aminotransferase; **1.1.1.237**: hydroxyphenylpyruvate reductase; **6.2.1.12**: 4-coumarate--CoA ligase; **3.1.2.23**: 4-hydroxybenzoyl-CoA thioesterase; **UbiC**: chorismate lyase; **UbiA/Coq2**: 4-hydroxybenzoate polyprenyltransferase; **UbiD/UbiX**: 3-octaprenyl-4-hydroxybenzoate carboxy-lyase; **UbiB**: ubiquinone biosynthesis protein; **UbiG** (EC:2.1.1.222): 2-polyprenyl-6-hydroxyphenyl methylase; **UbiH/Coq6**: 2-octaprenyl-6-methoxyphenol hydroxylase; **UbiE/Coq5**: ubiquinone biosynthesis methyltransferase; **UbiF/Coq7**: 2-octaprenyl-3-methyl-6-methoxy-1,4-benzoquinol hydroxylase; **UbiG/Coq3** (EC:2.1.1.64/EC:2.1.1.114): 3-demethylubiquinol 3-O-methyltransferase/hexaprenyldihydroxybenzoate methyltransferase.

Most of the genes responsible for this biosynthetic pathway from tyrosine are present in SHTs and RTs, however the first steps of this route are still not well characterized in related species (Figure 8). As concerns the route from chorismate, the enzyme UbiC (EC:4.1.3.40), which catalyzes the conversion of chorismate into 4-hydroxybenzoate, was identified only in the SHTs from the *Strigomonas* genus. In symbionts from this genus, we found this route with chorismate as precursor, however UbiC was not identified. Most bacteria from the Alcaligenaceae family have all the genes for ubiquinone production from chorismate while *Taylorella* spp. have some missing steps (Figure S1). The genes identified in all SHTs and RTs are also present in *Trypanosoma* and *Leishmania* spp. (Figure S1).

The presence of UbiC only in SHTs from the *Strigomonas* genus and of ubiquinone biosynthetic pathway only in their symbionts and not in other TPEs may indicate a higher production of ubiquinone in the *Strigomonas* host/symbiont system. As discussed in detail below in Section 3.4 (Phylogenetic Analyses), the gene *ubi*C is closely related to those described in proteobacteria.

### 3.4. Phylogenetic analyses

In trypanosomatids, most genes involved in the synthesis of vitamins are either of eukaryotic or of betaproteobacterial origin. In most cases, vitamin production benefits from the participation of the symbiotic bacterium whose genes are sister groups of the corresponding sequences described in *Bordetella* spp. and *Achromobacter* spp., both Betaproteobacteria that belong to the Alcaligenaceae family, as previously indicated for the heme biosynthesis genes [22]. As shown before in the whole genome analyses of these symbionts [24], the TPE genes involved in the synthesis of vitamins and the corresponding betaproteobacterial genes represent a monophyletic branch supported by bootstrap values close to 100 while they are distant from the equivalent genes in Alpha- and Gammaproteobacteria. The phylogenetic analyses of the trypanosomatid host genes were carried out using the ML and NJ methods, which gave similar trees thus reinforcing the obtained results. Most genes were found to be of eukaryotic origin while three genes may have been transferred from bacterial groups to the trypanosomatid hosts (Table S1).

**Figure 9:**
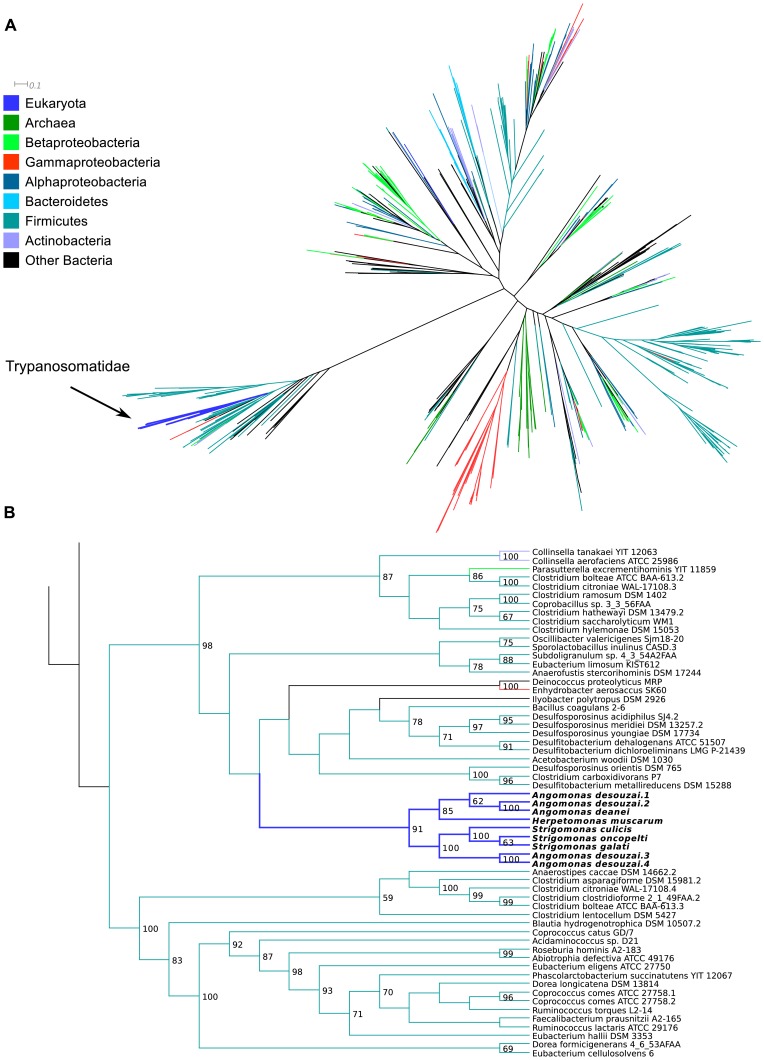
Maximum likelihood phylogenetic tree of ketopantoate reductase (EC:1.1.1.169). **A -**overall tree, colored according to taxonomic affiliation of each taxon, as per the legend on the left; distance bar only applies to panel A. **B –** details of the region of the tree where the Trypanosomatidae are placed. Values on nodes represent bootstrap support (only 50 or greater shown). Panel B is meant to only represent the branching patterns and do not portray estimated distances between sequences.

**3.4.1. Possible horizontal gene transfer (HGT) from Firmicutes to trypanosomatids.** The gene codifying for ketopantoate reductase (EC:1.1.1.169), involved in the synthesis of pantothenic acid (Figure 2), is present in the SHTs and in the genome of *H. muscarum* whereas it is absent in the TPE genomes. This gene is especially interesting due to the fact that all other steps for the synthesis of pantothenic acid are performed by enzymes coded by endosymbiont genes, including the enzymes necessary to synthesize the precursor α-ketoisovalerate. It is neither of proteobacterial nor of eukaryotic descent. With a high bootstrap support of 98 in the ML tree (85 in the NJ), its phylogeny indicates that it has been transferred to the SHTs and to *Herpetomonas* – or more probably to a common ancestor of these – from bacteria of the Firmicutes phylum (Figures 9 and S2).

It is interesting to note that the part of the phylogenetic tree containing the trypanosomatid gene, although mostly composed of Firmicutes, also includes genes of a few bacteria from other phyla interspersed amongst the Firmicutes genes. The phylum Firmicutes is divided in three major clades, with the clade containing the trypanosomatid genes separated from the other groups by a long branch (Figure 9). This could be due to different reasons: either the gene for EC:1.1.1.169 presents high evolutionary rates, leading to the long branch and low bootstrap values at deeper nodes of the tree and consequently to a difficulty in placing organisms in the tree; or there are multiple paralogs present in the tree due to ancient duplications. Our data do not permit to definitely distinguish between these two alternatives, although the much higher bootstrap support values at higher levels of the tree suggest the former.

**Figure 10:**
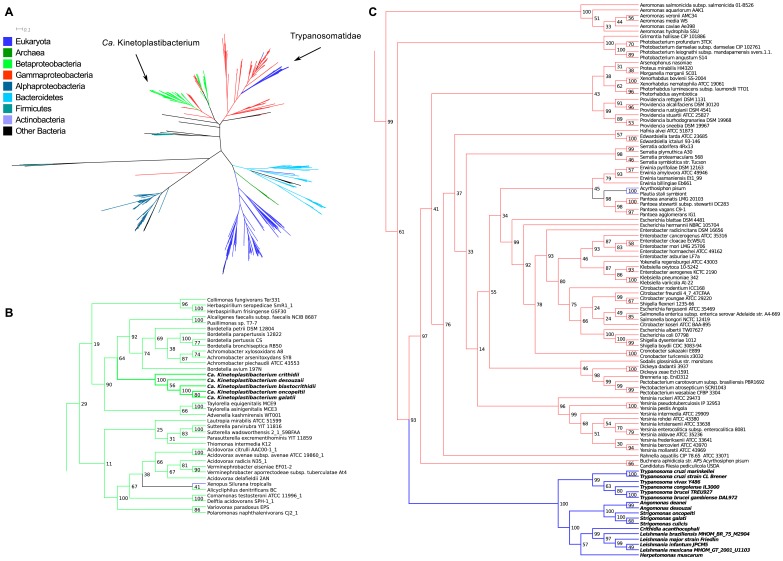
Maximum likelihood phylogenetic tree of nicotinate phosphoribosyltransferase (EC:2.4.2.11). **A –**overall tree, colored according to taxonomic affiliation of each taxon, as per the legend on the left; distance bar only applies to panel A. **B –** details of the region of the tree where the *Ca*. Kinetoplastibacterium spp. are placed. **C –** details of the region of the tree where the Trypanosomatidae are placed. Values on nodes represent bootstrap support (only 50 or greater shown). Panels B and C are meant to only represent the branching patterns and do not portray estimated distances between sequences.

**3.4.2. Possible HGT from Gammaproteobacteria to trypanosomatids.** The gene for nicotinate phosphoribosyltransferase (EC:2.4.2.11), involved in the salvage pathway of nicotinic acid (Figure 6), is present in the SHT, RT, and TPE genomes. The trypanosomatids form a monophyletic group (bootstrap support of 100), and group within the Gammaproteobacteria with a high bootstrap support value of 93 in the ML tree (91 in the NJ, Figures 10 and S3). They are far from the other eukaryotes in the tree and overall form a monophyletic clade with moderate (66) and high (91) support values in the ML and NJ trees, respectively. The few other eukaryotes are placed within other bacterial groups; one such example concerns *Entamoeba* spp. placed within the Bacteroidetes (high support value of 95 and 81 in the ML and NJ trees, respectively). The TPEs group within the Alcaligenaceae family with high bootstrap support values of 90 and 99 (ML and NJ trees, respectively).

**Figure 11:**
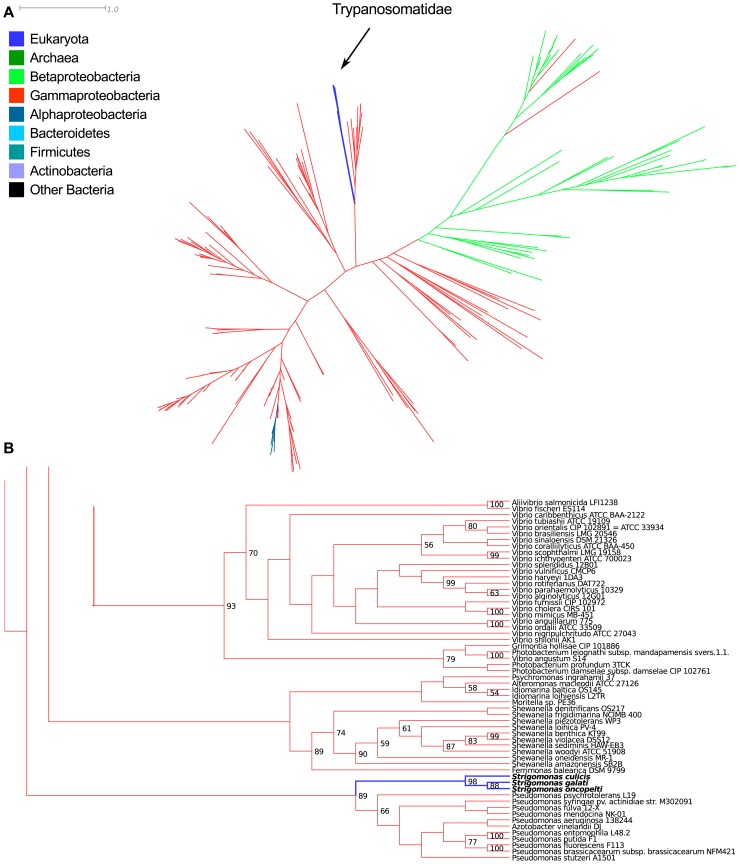
Maximum likelihood phylogenetic tree of UbiC (EC:4.1.3.40). **A –**overall tree, colored according to taxonomic affiliation of each taxon, as per the legend on the left; distance bar only applies to panel A. B – details of the region of the tree where the Trypanosomatidae are placed. Values on nodes represent bootstrap support (only 50 or greater shown). Panel B is meant to only represent the branching patterns and do not portray estimated distances between sequences.

The gene for UbiC (EC:4.1.3.40), involved in the synthesis of ubiquinone (Figure 8), is present only in the SHTs of the *Strigomonas* genus, but is absent from the genome of any *Angomonas*, TPE, or RT. The three *Strigomonas* form a monophyletic group, and are placed as the sister group of the genus *Pseudomonas* (Gammaproteobacteria) with a high bootstrap support value of 89 (Figures 11 and S4). The overall tree for UbiC contains almost only Beta- and Gammaproteobacteria, with a few Alphaproteobacteria of the *Bartonella* genus present within the Gammaproteobacteria.

**3.4.3. Genomic context and possible acquisition of HGTs.** These potentially transferred genes are mainly located in contigs presenting the typical trypanosomatid architecture of long stretches of genes in the same orientation (Figure S5). One such example is the upstream gene of ketopantoate reductase (EC:1.1.1.169) which is the one codifying for pyruvate kinase (EC:2.7.1.40), which is involved in the glycolytic pathway. This same genomic context was found in the previously sequenced strain of *A. deanei* [38]. In addition to that, the presence/absence of these three genes (codifying for EC:1.1.1.169, EC:2.4.2.11, EC:4.1.3.40) in the previously sequenced genomes of *A. deanei* and *S. culicis* are in agreement with the findings herein presented [38]. The GC percent (Figure S5) and sequencing coverage (Table S1) analyses also show that these genes present statistics typical of other genes from these organisms. The HGT genes analyzed show a codon usage consistent with that of about 125 other nuclear genes of the trypanosomatid based on the codon adaptation index and the correspondence analysis performed using a TPE gene as negative control (Figure S6).

The association of the betaproteobacterial symbionts and of the trypanosomatid hosts is very ancient, estimated to have occurred in the late Cretaceous [66], [13] and to have perpetuated since by vertical transmission. No dating or any other kind of information is available about the acquisition of genes for vitamin synthesis of bacterial origin by trypanosomatids. It may be presumed, as is the case for similar instances of genes involved in the synthesis of heme or amino acids, that lateral gene transfer occurred in a common ancestor of several extant Trypanosomatidae clades, being subsequently lost in those where it was no longer necessary for the metabolism of the organism [22], [23]. Since the gene for the enzyme EC:1.1.1.169 is identified as being in a monophyletic group with the SHTs and *Herpetomonas* with a high bootstrap value of 91, this indicates that it was acquired by a common ancestor of these flagellates, and that other related genera and species might have been involved. However, the precise point in the tree of the family, or higher taxonomic category, where this gene was acquired remains obscure. Therefore, more studies are needed on the composition of the genes involved in the synthesis of vitamins, in more distantly related, non-parasitic Kinetoplastida, in order to try to elucidate this point of their genomic evolution.

## Conclusions

**Figure 12:**
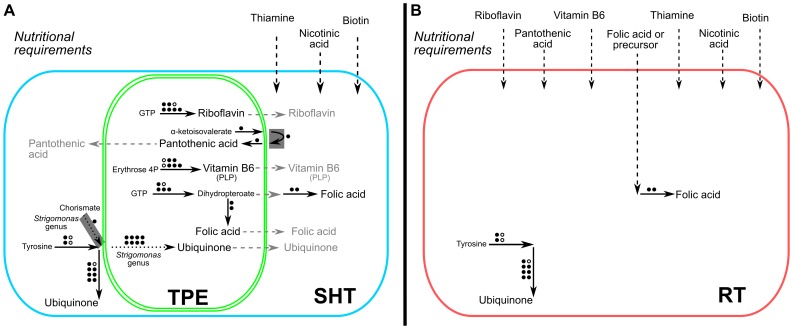
Overview of the biosynthetic pathways of essential vitamins and cofactors in trypanosomatids. Dashed arrows: metabolite import/exchange; dotted arrows: reaction present in only some of the organisms analyzed; solid arrows: other reactions (circles on the top of the arrows indicate number of steps and fulfilled circles indicate presence of enzyme); arrows surrounded by a gray box: enzymes possibly acquired through horizontal transfer from Bacteria to trypanosomatids (see main text). **A -** Contribution of SHTs and TPEs based on the analysis of gene content in the genomes of *A. deanei*, *A. desouzai*, *S. culicis*, *S. oncopelti*, *S. galati* and respective endosymbionts. **B -** Biochemical capability of trypanosomatids without symbionts based on the analysis of genomic data of *H. muscarum*, *C. acanthocephali* and *L. major*.

The results obtained in this work are in agreement with earlier nutritional studies [3], [5], [9], [19] which indicated that trypanosomatids require seven vitamins in the culture media: folic and pantothenic acid, biotin, vitamin B6, riboflavin, thiamine, and nicotinic acid (Figure 12). As shown in the present study, this is related to the fact that such protozoa lack the complete set of genes that codify for the enzymes involved in these essential biosynthetic pathways. However, this nutritional requirement does not apply to trypanosomatids carrying a cytoplasmic endosymbiont. SHTs have the necessary enzymes to produce most vitamins, with the exception of thiamine, biotin, and nicotinic acid, which represent absolute nutritional requirements for trypanosomatids in general. Most of the genes related to the synthesis of riboflavin, vitamin B_6_, and folic acid were identified only in the symbiont genomes. This indicates the presence of complete biosynthetic routes in the TPEs with an exchange of metabolites between host and bacterium in the extremities of the pathway, *i.e.* precursors and end products. On the other hand, the same is not observed in the synthesis of pantothenic acid, as suggested by our analyses. This pathway might have a more intricate participation of both partners in intermediate steps. SHTs and TPEs are able to perform the conversion of the vitamins riboflavin and pantothenic acid into the essential metabolites FAD and CoA, which indicates that possibly the symbiont enhances the production of these metabolites which may be controlled by the host in a way that is not yet fully elucidated.

According to the phylogenetic analyses, some genes coding for the enzymes involved in the biosynthetic and salvage pathways of vitamins and cofactors are in the host genome and are of eukaryotic origin, while most genes are localized in the genomes of the symbionts and are of betaproteobacterial ancestry. On the other hand, three genes were possibly transferred from bacteria to the trypanosomatid nuclei. Such is the case of the ketopantoate reductase gene (EC:1.1.1.169) involved in the *de novo* biosynthesis of pantothenic acid, which was probably transferred from a Firmicutes bacterium to an ancestor of the SHT host and of *H. muscarum*. The two other sequences may have been acquired from Gammaproteobacteria: nicotinate phosphoribosyltransferase (EC:2.4.2.11), which is involved in the salvage pathway of nicotic acid, and UbiC (EC:4.1.3.40), which is involved in the synthesis of ubiquinone.

Taken together, the nutritional data and our genomic analysis show that SHTs are autotrophic for riboflavin, pantothenic acid, vitamin B_6_, and folic acid [6], [7], [9], [21]. As a result, we can assume that the shared participation of the trypanosomatid host and of its symbiont in the synthesis of vitamins evidences an extensive metabolic exchange between both partners, at the extremities of the pathways or maybe even at intermediate steps, and that this exchange has an essential role in the maintenance of this mutualistic association.

## Supporting Information

Figure S1 **Comparative analysis of trypanosomatids and bacteria from the Alcaligenaceae family and the species analyzed in the present study.** Green squares indicate presence while gray ones indicate absence of the enzyme (column) in the respective organism (line). Information of trypanosomatids and bacteria other than the ones analyzed in the present study are from KEGG.
(PDF)

Figure S2 **Neighbor joining phylogenetic tree of ketopantoate reductase (EC:1.1.1.169). A -** overall tree, colored according to taxonomic affiliation of each taxon, as per the legend on the left; distance bar only applies to panel A. **B –** details of the region of the tree where the Trypanosomatidae are placed. Values on nodes represent bootstrap support (only 50 or greater shown). Panel B is meant to only represent the branching patterns and do not portray estimated distances between sequences.
(PNG)

Figure S3 **Neighbor joining phylogenetic tree of nicotinate phosphoribosyltransferase (EC:2.4.2.11). A –** overall tree, colored according to taxonomic affiliation of each taxon, as per the legend on the left; distance bar only applies to panel A. **B –** details of the region of the tree where the *Ca*. Kinetoplastibacterium spp. are placed. **C –** details of the region of the tree where the Trypanosomatidae are placed. Values on nodes represent bootstrap support (only 50 or greater shown). Panels B and C are meant to only represent the branching patterns and do not portray estimated distances between sequences.
(PNG)

Figure S4 **Neighbor joining phylogenetic tree of UbiC (EC:4.1.3.40). A –** overall tree, colored according to taxonomic affiliation of each taxon, as per the legend on the left; distance bar only applies to panel A. **B** – details of the region of the tree where the Trypanosomatidae are placed. Values on nodes represent bootstrap support (only 50 or greater shown). Panel B is meant to only represent the branching patterns and do not portray estimated distances between sequences.
(PNG)

Figure S5 **Genomic context for candidate HGT genes in the Trypanosomatidae analyzed in this work.** Arrows show TBLASTN alignments of the genome against UniRef100 and KEGG proteins, as displayed by GBrowse and edited for clarity of presentation. The gene currently in focus is colored black. Coordinates are in kilobases.
(PNG)

Figure S6 **Codon adaptation index and correspondence analysis of codon usage for candidate HGT genes.** Red: candidate HGT genes of the Trypanosomatidae analyzed in this work and the negative control which is the endosymbiont gene BCUe_0001. Codon adaptation index for *A. deanei* genes (A), for *A. desouzai* genes (B) and for S. *galati* (C). Correspondence analysis of codon usage for *A. deanei* genes (D), for *A. desouzai* genes (E) and for S. *galati* (F).
(PDF)

Table S1 **Summary of the phylogenetic and sequencing coverage analyses of the candidate HGT genes.**
(PDF)

Table S2 **Trypanosomatidae genes characterized in this study.**
(XLS)

Table S3 ***Ca.*** **Kinetoplastibacterium genes analyzed in this study.**
(XLS)
