# Supplementary figures and images for: Biosynthesis of Vitamins and Cofactors in Bacterium-Harbouring Trypanosomatids Depends on the Symbiotic Association as Revealed by Genomic Analyses
Source: PLoS One. 2013 Nov 19;8(11):e79786. doi: 10.1371/journal.pone.0079786 (PMC3833962; doi:10.1371/journal.pone.0079786)

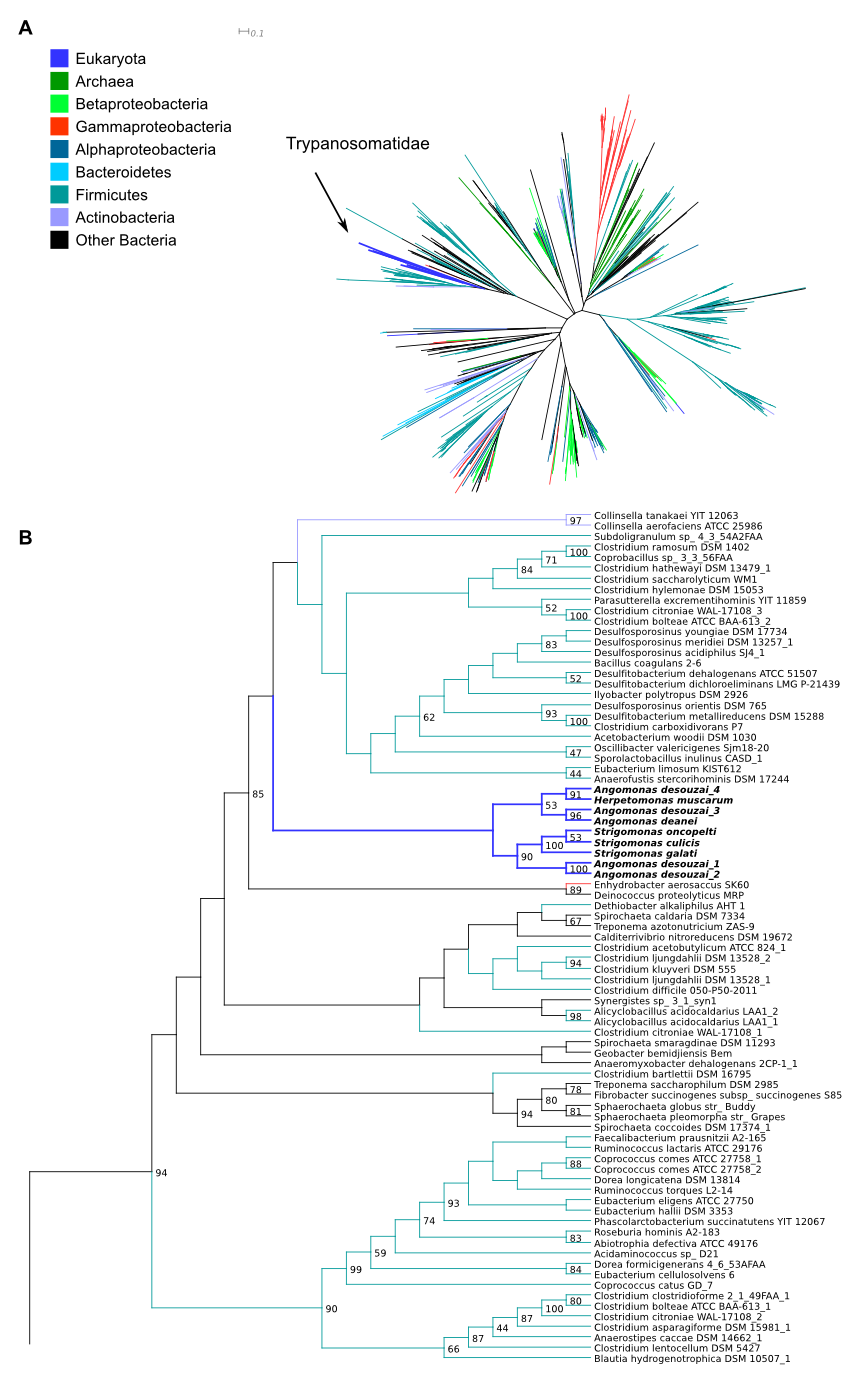

Supplement: Figure S2 — Neighbor joining phylogenetic tree of ketopantoate reductase (EC:1.1.1.169). A - overall tree, colored according to taxonomic affiliation of each taxon, as per the legend on the left; distance bar only applies to panel A. B – details of the region of the tree where the Trypanosomatidae are placed. Values on nodes represent bootstrap support (only 50 or greater shown). Panel B is meant to only represent the branching patterns and do not portray estimated distances between sequences. (PNG) [file pone.0079786.s002.png]

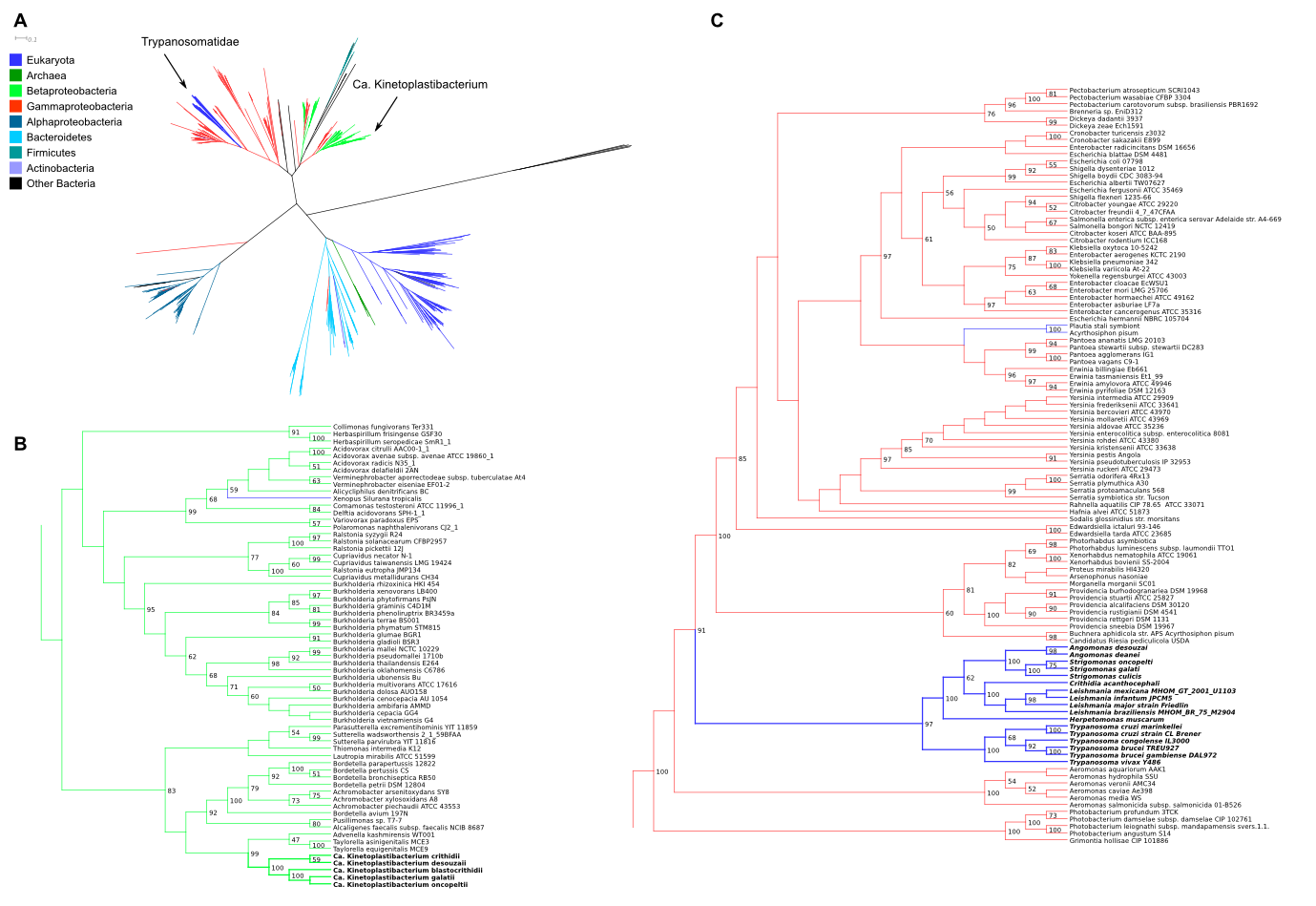

Supplement: Figure S3 — Neighbor joining phylogenetic tree of nicotinate phosphoribosyltransferase (EC:2.4.2.11). A – overall tree, colored according to taxonomic affiliation of each taxon, as per the legend on the left; distance bar only applies to panel A. B – details of the region of the tree where the Ca. Kinetoplastibacterium spp. are placed. C – details of the region of the tree where the Trypanosomatidae are placed. Values on nodes represent bootstrap support (only 50 or greater shown). Panels B and C are meant to only represent the branching patterns and do not portray estimated distances between sequences. (PNG) [file pone.0079786.s003.png]

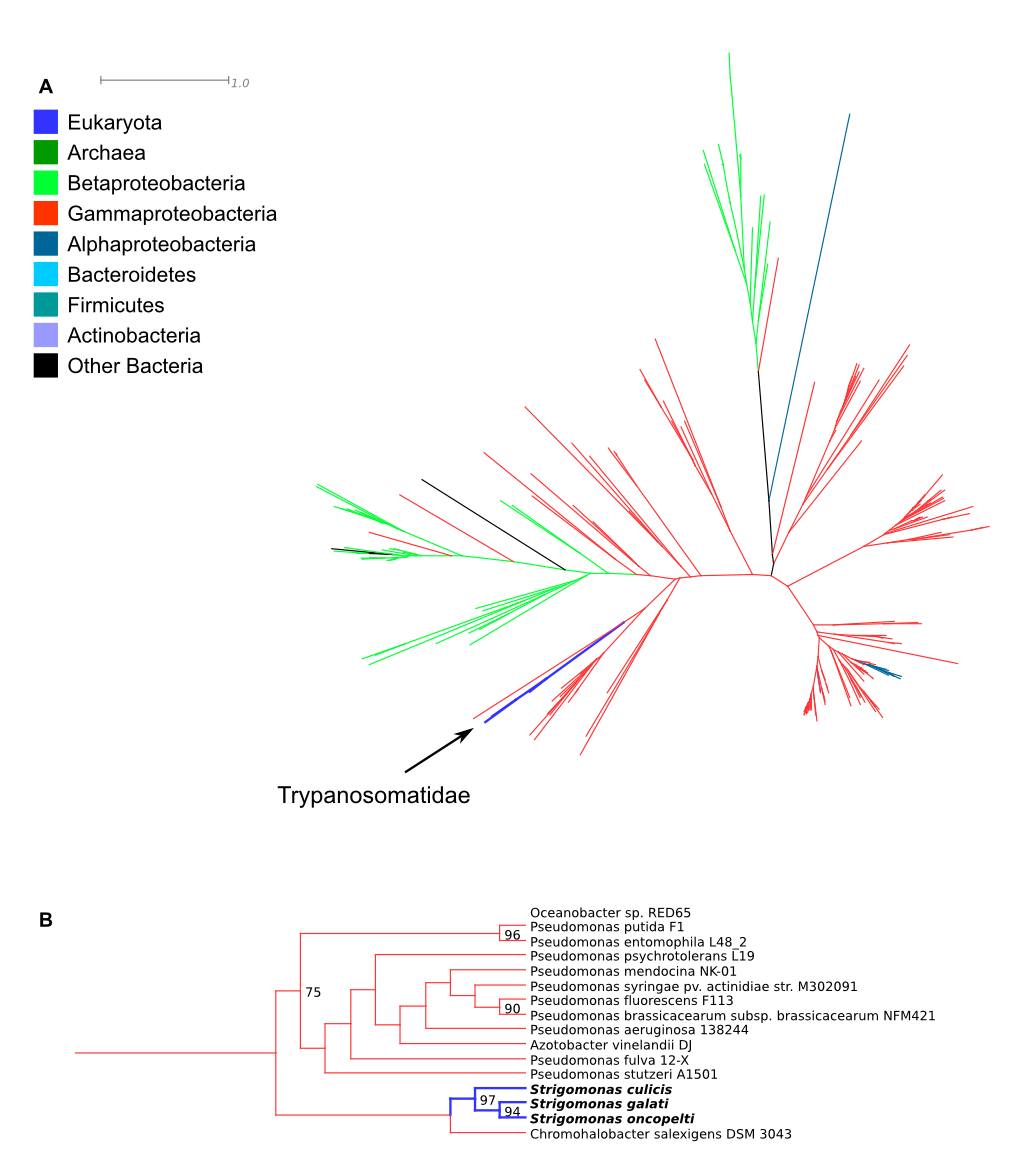

Supplement: Figure S4 — Neighbor joining phylogenetic tree of UbiC (EC:4.1.3.40). A – overall tree, colored according to taxonomic affiliation of each taxon, as per the legend on the left; distance bar only applies to panel A. B – details of the region of the tree where the Trypanosomatidae are placed. Values on nodes represent bootstrap support (only 50 or greater shown). Panel B is meant to only represent the branching patterns and do not portray estimated distances between sequences. (PNG) [file pone.0079786.s004.png]

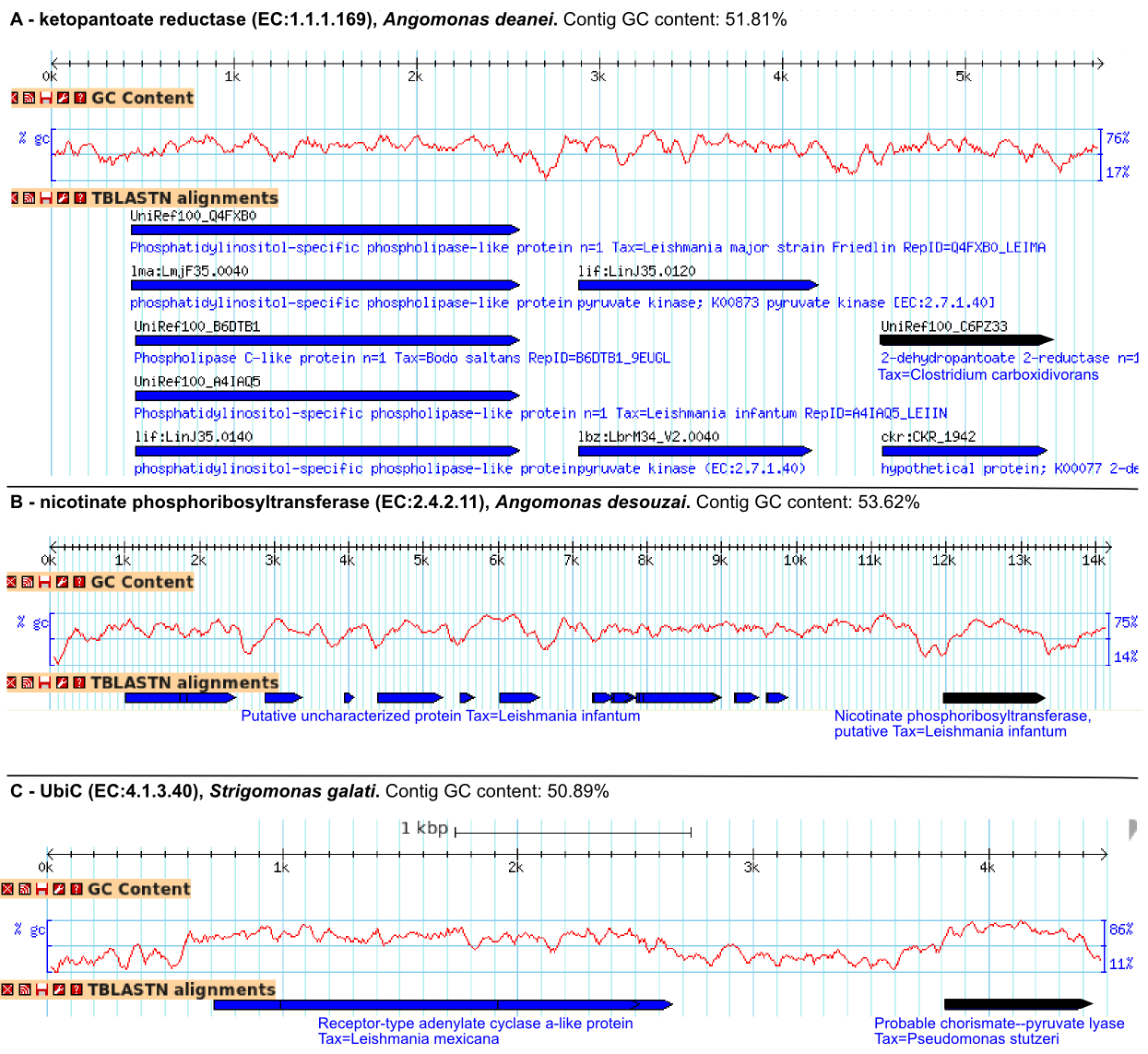

Supplement: Figure S5 — Genomic context for candidate HGT genes in the Trypanosomatidae analyzed in this work. Arrows show TBLASTN alignments of the genome against UniRef100 and KEGG proteins, as displayed by GBrowse and edited for clarity of presentation. The gene currently in focus is colored black. Coordinates are in kilobases. (PNG) [file pone.0079786.s005.png]
