# Supplementary material for: Biosynthesis of Vitamins and Cofactors in Bacterium-Harbouring Trypanosomatids Depends on the Symbiotic Association as Revealed by Genomic Analyses
Source: PLoS One. 2013 Nov 19;8(11):e79786. doi: 10.1371/journal.pone.0079786 (PMC3833962; doi:10.1371/journal.pone.0079786)
Supplement: Figure S6 — Codon adaptation index and correspondence analysis of codon usage for candidate HGT genes. Red: candidate HGT genes of the Trypanosomatidae analyzed in this work and the negative control which is the endosymbiont gene BCUe_0001. Codon adaptation index for A. deanei genes (A), for A. desouzai genes (B) and for S. galati (C). Correspondence analysis of codon usage for A. deanei genes (D), for A. desouzai genes (E) and for S. galati (F). (PDF) [file pone.0079786.s006.pdf]

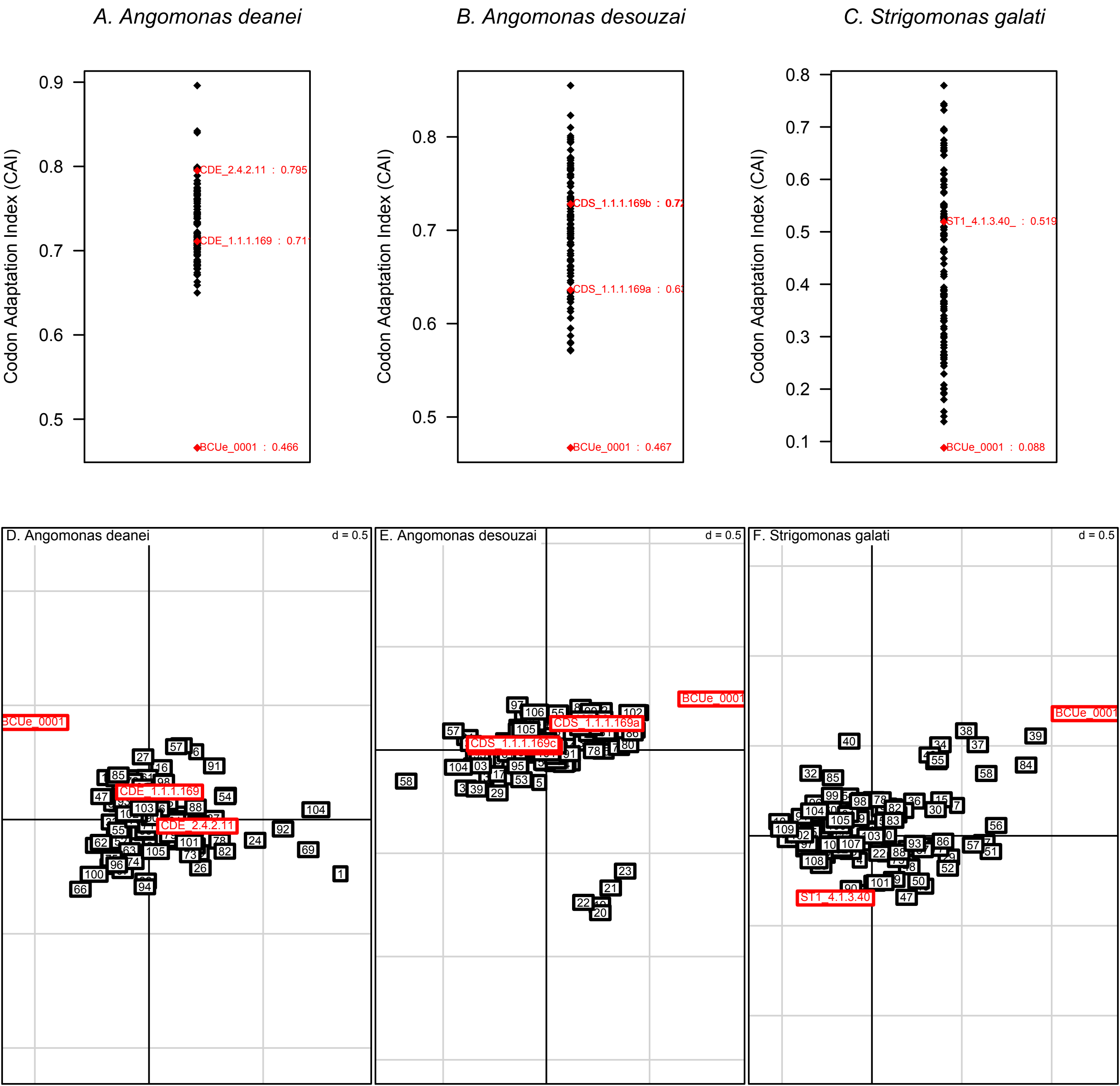

A. Angomonas deanei
B. Angomonas desouzai
C. Strigomonas galati
Codon Adaptation Index (CAI)
CDE_2.4.2.11 : 0.795
CDE_1.1.1.169 : 0.71
BCUe_0001 : 0.466
CDS_1.1.1.169b : 0.72
CDS_1.1.1.169a : 0.63
BCUe_0001 : 0.467
ST1_4.1.3.40_ : 0.519
BCUe_0001 : 0.088
D. Angomonas deanei
E. Angomonas desouzai
F. Strigomonas galati
d = 0.5
