# Supplementary material for: Biosynthesis of Vitamins and Cofactors in Bacterium-Harbouring Trypanosomatids Depends on the Symbiotic Association as Revealed by Genomic Analyses
Source: PLoS One. 2013 Nov 19;8(11):e79786. doi: 10.1371/journal.pone.0079786 (PMC3833962; doi:10.1371/journal.pone.0079786)
Supplement: Table S1 — Summary of the phylogenetic and sequencing coverage analyses of the candidate HGT genes. (PDF) [file pone.0079786.s007.pdf]

Summary phylogenetic and sequencing coverage analyses

EC number	Enzyme name	Pathway	Nb Sequences	Nb Sites	# Distinct Alignment Patterns	Organisms	ML – Cluster / Sister group trypanosomatids	NJ – Cluster / Sister group trypanosomatids	Cluster / Sister group TPEs	Figure	Average genomic coverage	Average contig coverage	Average gene coverage	Genome*
1.1.1.169	2-dehydropantoate 2-reductase	Pantothenate	607	1123	962	All SHTs and Herpetomonas, but not the other RTs.	They group within Firmicutes (BS=98).	They group within Firmicutes and a few other groups of bacteria (BS=85).	-	9	23x	18x	18x	*A. deanei*
2.4.2.11	nicotinate phosphoribosyltr ansferase	Nicotinate	630	1050	1010	All SHTs, RTs and TPEs	Trypanosomatid clade (BS=100) clusters within the Gammaproteobacteria (BS=93).	Trypanosomatid clade (BS=97) cluster within the Gammaproteobacteria (BS=91).	ML: Group with Alcaligenaceae (BS=90). NJ: Group with *Taylorella* and *Advenella* spp. (BS=99), and with the Alcaligenaceae (low BS).	10	24x	22x	27x	*A. desouzai*
4.1.3.40	chorismate lyase	Ubiquinone	217	389	372	SHTs from Strigomonas genus	Strigomonas clade (BS=98) very similar to Pseudomonas, clusters within Gammaproteobacteria (BS=89), although this gene seems to diverge quite fast, making the identification of putative orthologs difficult.	Strigomonas clade (BS=97) clusters within Gammaproteobacteria (low BS).	-	11	28x	14x	14x	*S. galati*

* Genome, contig, and gene average sequencing coverages were calculated for the organism indicated in the “Genome” column.
