# Supplementary material for: Biosynthesis of Vitamins and Cofactors in Bacterium-Harbouring Trypanosomatids Depends on the Symbiotic Association as Revealed by Genomic Analyses
Source: PLoS One. 2013 Nov 19;8(11):e79786. doi: 10.1371/journal.pone.0079786 (PMC3833962; doi:10.1371/journal.pone.0079786)
Supplement: Figure S1 — Comparative analysis of trypanosomatids and bacteria from the Alcaligenaceae family and the species analyzed in the present study. Green squares indicate presence while gray ones indicate absence of the enzyme (column) in the respective organism (line). Information of trypanosomatids and bacteria other than the ones analyzed in the present study are from KEGG. (PDF) [file pone.0079786.s001.pdf]

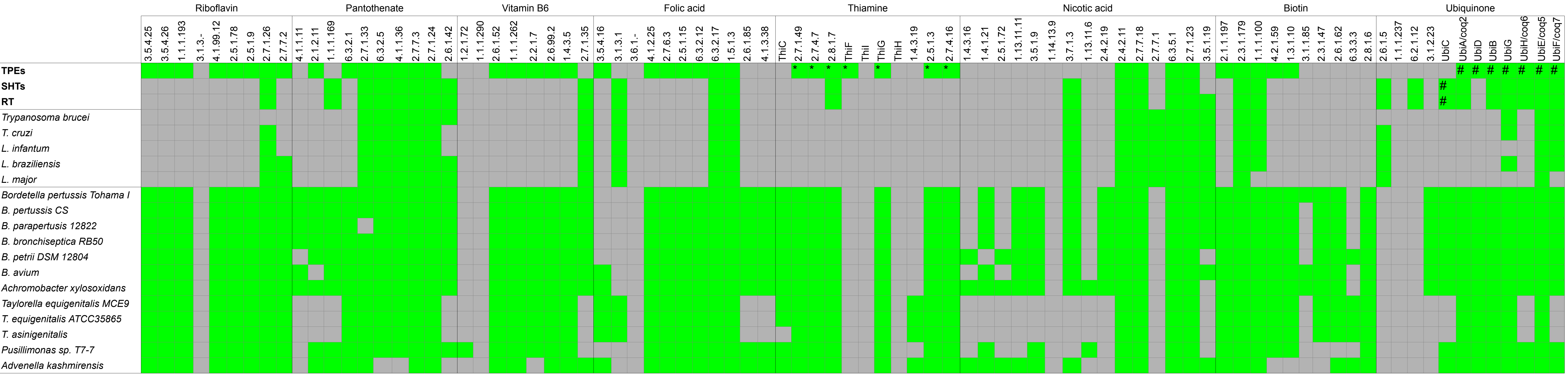

*Angomonas

#Strigomonas
